# NanoPack: visualizing and processing long-read sequencing data

**DOI:** 10.1093/bioinformatics/bty149

**Published:** 2018-03-14

**Authors:** Wouter De Coster, Svenn D’Hert, Darrin T Schultz, Marc Cruts, Christine Van Broeckhoven

**Affiliations:** 1 Neurodegenerative Brain Diseases Group, VIB & University of Antwerp, Antwerp, Belgium; 2 Bioinformatics, Neuromics Support Facility, Center for Molecular Neurology, VIB & University of Antwerp, Antwerp, Belgium; 3 Department of Biomolecular Engineering and Bioinformatics, University of California Santa Cruz, Santa Cruz, CA, USA

## Abstract

**Summary:**

Here we describe NanoPack, a set of tools developed for visualization and processing of long-read sequencing data from Oxford Nanopore Technologies and Pacific Biosciences.

**Availability and implementation:**

The NanoPack tools are written in Python3 and released under the GNU GPL3.0 License. The source code can be found at https://github.com/wdecoster/nanopack, together with links to separate scripts and their documentation. The scripts are compatible with Linux, Mac OS and the MS Windows 10 subsystem for Linux and are available as a graphical user interface, a web service at http://nanoplot.bioinf.be and command line tools.

**Supplementary information:**

Supplementary data are available at *Bioinformatics* online.

## 1 Introduction

The dominant sequencing by synthesis technology is characterized by sequencing a fixed short read length template (50–300 bp) with high accuracy (error rate <1%) (Goodwin *et al.*, 2016). In contrast, long-read sequencing methods from Oxford Nanopore Technologies (ONT) and Pacific Biosciences routinely achieve read lengths of 10 kb, with a long tail of up to 1.2 Megabases for ONT (unpublished results). These long reads come with a tradeoff of lower accuracy of about 85–95% (Giordano *et al.*, 2017; Jain *et al.*, 2017, 2018). It is evident that these characteristics make many existing Illumina-tailored QC tools, such as FastQC (Babraham Bioinformatics 2010, https://www.bioinformatics.babraham.ac.uk/projects/fastqc/), suboptimal for long-read technologies. NanoPack, a set of Python scripts for visualizing and processing long-read sequencing data, was developed to partially bridge this gap. Earlier tools such as poretools (Loman and Quinlan, 2014), poRe (Watson *et al.*, 2015) and IONiseR (Smith, 2017) mainly focused on feature extraction from the older fast5 file formats, and alternative tools such as pycoQC (Leger, 2017) and minion_qc (Lanfear, n.d. https://github.com/roblanf/minion_qc) do not offer the same flexibility and options as NanoPack. The plotting style from the pauvre tool (Schultz, n.d. https://github.com/conchoecia/pauvre) got incorporated in NanoPack (Supplementary Fig. S3).

## 2 Software description

### 2.1 Installation and dependencies

NanoPack and individual scripts are available through the public software repositories PyPI using pip and bioconda through conda (Dale *et al.*, 2017). The scripts build on a number of third party Python modules: matplotlib (Hunter, 2007), pysam (Heger, 2009; Li *et al.*, 2009; https://github.com/pysam-developers/pysam), pandas (McKinney, 2011), numpy (Walt *et al.*, 2011), seaborn (Waskom *et al.*, 2017) and biopython (Cock *et al*., 2009).

### 2.2 Scripts for statistic evaluation and visualization

**Fig. 1.**
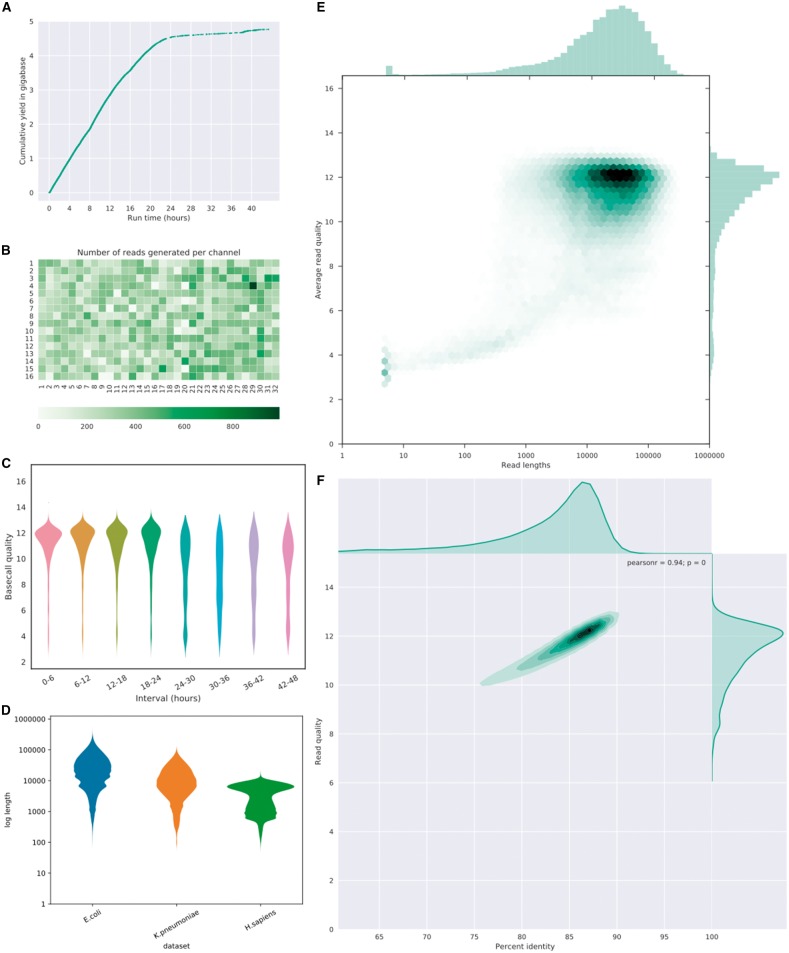
Examples of plots of NanoPlot and NanoComp. (**A**) Cumulative yield plot (**B**) Flow cell activity heatmap showing number of reads per channel. (**C**) Violin plots comparing base call quality over time. (**D**) NanoComp plot comparing log transformed read lengths of the *E.coli* dataset with a *K.pneumoniae* and human dataset. (**E**) Bivariate plot of log transformed read length against base call quality with hexagonal bins and marginal histograms. (**F**) Bivariate plot of base call quality against percent identity with a kernel density estimate and marginal density plots

NanoStat produces a comprehensive statistical data summary (Supplementary Table S2). NanoPlot and NanoComp produce informative QC graphs displaying multiple aspects of sequencing data (Fig. 1, Supplementary Table S1) and accept input data in (compressed) fastq or fasta format, bam and (compressed) albacore summary files or multiple files of the same type.

All plots and summary statistics are combined in an html report. Because long and variable read lengths may be challenging to interpret on a linear axis, there is also an option to plot the read lengths on a log scale. Plots can be produced in standard image file formats including png, jpg, pdf and svg. NanoPlot produces read length histograms, cumulative yield plots, violin plots of read length and quality over time and bivariate plots comparing the relationship between read lengths, quality scores, reference identity and read mapping quality. Better insight in big datasets can be obtained using bivariate plots with a 2D kernel density estimation or hexagonal bins (Fig. 1E and F, Supplementary Fig. S3). Optional arguments include random down sampling of reads and removing all reads above a length cutoff or below a quality cutoff. Data from a multiplexed experiment in albacore summary format can be separated, resulting in plots and statistics per barcode. NanoComp performs comparison across barcodes or experiments of read length and quality distributions, number of reads, throughput and reference identity.

### 2.3 Scripts for data processing

NanoFilt and NanoLyse were developed for processing reads in streaming applications and therefore have a minimal memory footprint and can be integrated in existing pipelines prior to alignment. NanoFilt is a tool for read filtering and trimming. Filtering can be performed based on mean read quality, read length and mean GC content. Trimming can be done with a user-specified number of nucleotides from either read ends. NanoLyse is a tool for rapid removal of contaminant DNA, using the Minimap2 aligner through the mappy Python binding (Li, 2017). A typical application would be the removal of the lambda phage control DNA fragment supplied by ONT, for which the reference sequence is included in the package. However, this approach may lead to unwanted loss of reads from regions highly homologous to the lambda phage genome.

## 3 Examples and discussion

The NanoPlot and NanoComp examples (Fig. 1) are based on an ONT *Escherichia coli* dataset from an ultra-long-read protocol sequenced on an R9.4 MinION flow cell (Quick and Loman, 2017; http://lab.loman.net/2017/03/09/ultrareads-for-nanopore/) generating 150 735 reads, base called using Albacore 2.0.2 and aligned to the *E.coli* reference genome using Minimap2 (Li, 2017). The cumulative yield (Fig. 1A) shows a lower efficiency when the flow cell wears out. A heat map of the physical layout of the MinION flow cell (Fig. 1B) highlights more productive channels and could potentially identifying suboptimal loading conditions, such as introduction of an air bubble. The mean base call quality per 6 h interval (Fig. 1C) shows a uniform high quality in the beginning, with lower quality reads after 24 h. In a bivariate plot comparing log transformed read lengths with their mean quality score (Fig. 1E) the majority of reads can be identified at lengths of 10 kb and quality scores of 12 by the color intensity of the hexagonal bins, with a subgroup of low-quality short reads. Plotting the mean quality against the per read percent reference identity (as a proxy for accuracy) (Fig. 1F) highlights a strong correlation, here with the number of reads plotted using a kernel density estimate. Additional examples from NanoPlot can be found in the supplementary information online, including standard and log transformed histograms, optionally with the N50 metric (Supplementary Figs S1 and S2) and a bivariate plot comparing effective read length with aligned read length (Supplementary Fig. S4), identifying reads which are only partially aligned to the reference genome.

The NanoComp plot (Fig. 1D) compares the log transformed read lengths of the same *E.coli* dataset to a *Klebsiella pneumoniae* (Wick *et al.*, 2017) and a human PromethION dataset (unpublished), clearly showing differences in the length profile with far longer reads in the *E.coli* dataset, standard read lengths in the library prep by ligation from *K.pneumoniae* and suboptimal read lengths from the human sample. Additional examples from NanoComp can be found in the supplementary information online, indicating that the *K.pneumoniae* library has both the highest yield (Supplementary Fig. S5) and on average higher quality scores (Supplementary Fig. S6) than both the human and *E.coli* dataset, but a comparable percent identity (Supplementary Fig. S7) with the human dataset.

## 4 Conclusion

NanoPack is a package of efficient Python scripts for visualization and processing of long-read sequencing data available on all major operating systems. Installation from the PyPI and bioconda public repositories is trivial, automatically taking care of dependencies. The plotting tools are flexible and customizable to the users need. Using a single NanoPlot or NanoComp command a full html report containing all summary statistics and plots can be prepared, and the software is easily accessible through the graphical user interface and web service, in addition to the command line scripts.

## Supplementary Material

Supplementary Data
