# Supplementary material for: NanoPack: visualizing and processing long-read sequencing data
Source: Bioinformatics. 2018 Mar 14;34(15):2666–9. doi: 10.1093/bioinformatics/bty149 (PMC6061794; doi:10.1093/bioinformatics/bty149)
Supplement: Supplementary Data [file bty149_nanopack_20180102_supplementary.docx]

# Supplementary information

| **Plot** | **input data** | | | | | **Options** |
| --- | --- | --- | --- | --- | --- | --- |
|  | Fastq | Fastq rich | Fastq minimal | Bam | Albacore Summary |  |
| Histogram of read length | x | x | x | x | x | N50 |
| Histogram of log transformed read length | x | x | x | x | x | N50 |
| Bivariate plot of length against base call quality | x | x |  | x | x | log transformation |
| Heatmap of reads per channel |  | x |  |  | x |  |
| Cumulative yield plot |  | x | x |  | x |  |
| Violin plot of read length over time |  | x | x |  | x |  |
| Violin plot of base call quality over time |  | x |  |  | x |  |
| Bivariate plot of aligned read length against sequenced read length |  |  |  | x |  |  |
| Bivariate plot of percent reference identity against read length |  |  |  | x |  | log transformation |
| Bivariate plot of percent reference identity against base call quality |  |  |  | x |  |  |
| Bivariate plot of mapping quality against read length |  |  |  | x |  | log transformation |
| Bivariate plot of mapping quality against basecall quality |  |  |  | x |  |  |

## Supplementary table 1: NanoPlot plots

## Supplementary table 2: NanoStat example output

| General statistics | |
| --- | --- |
| Number of reads: | 150,735 |
| Total bases: | 4,769,579,795 |
| Median read length: | 18,796.00 |
| Mean read length: | 31,642.15 |
| Readlength N50: | 61,411 |
| Top 5 read lengths and their average base call quality score | |
| Length: 763,488bp | Q: 11.64 |
| Length: 760,365bp | Q: 11.46 |
| Length: 744,361bp | Q: 10.38 |
| Length: 656,649bp | Q: 12.13 |
| Length: 628,176bp | Q: 11.96 |
| Top 5 average base call quality scores and their read lengths | |
| Length: 323bp | Q: 16.57 |
| Length: 29,090bp | Q: 14.1 |
| Length: 8,297bp | Q: 14.05 |
| Length: 1,233bp | Q: 13.94 |
| Length: 112,093bp | Q: 13.92 |
| Number of reads and fraction above quality cutoffs: | |
| Q5: | 143,721 (95.35%) |
| Q10: | 114,990 (76.29%) |
| Q15: | 1 (0.00%) |
| Q20: | 0 (0.00%) |
| Data produced using 506 active channels. | |

## Supplementary Figure S1: Histogram of read lengths


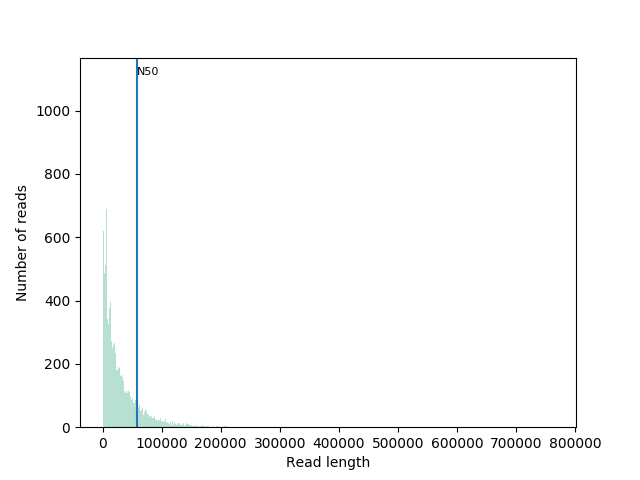


NanoPlot histogram of read lengths in bins of 100bp, showing the read length N50 value.

## Supplementary Figure S2: Histogram of log transformed read lengths


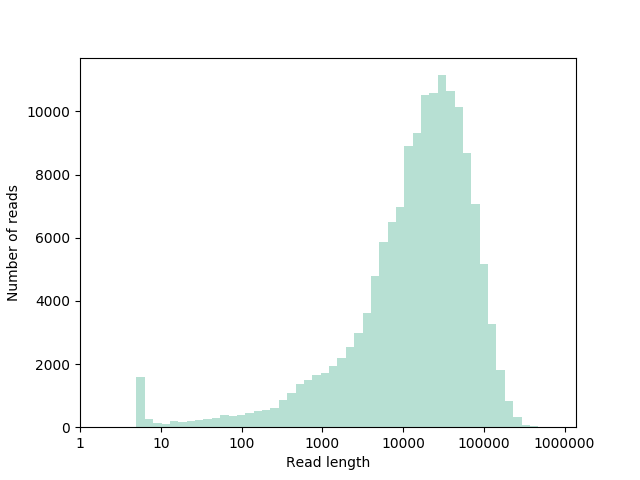


NanoPlot histogram of log transformed read lengths

## Supplementary Figure S3: Quality versus length plot in ‘pauvre’ style

##
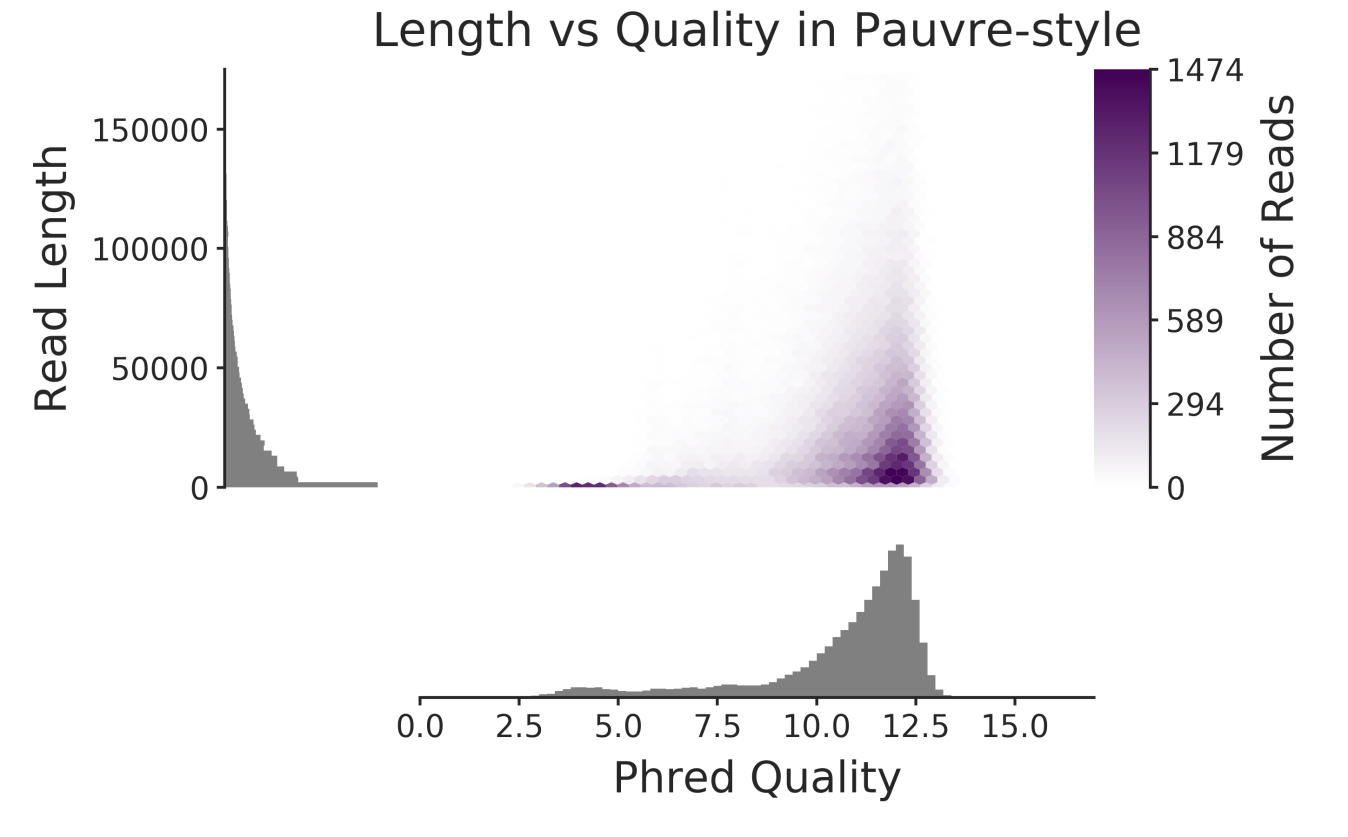


## NanoPlot bivariate plot of per read reads lengths against mean basecall quality score in pauvre style.

## Supplementary Figure S4: Bivariate plot of sequenced read lengths against aligned read length


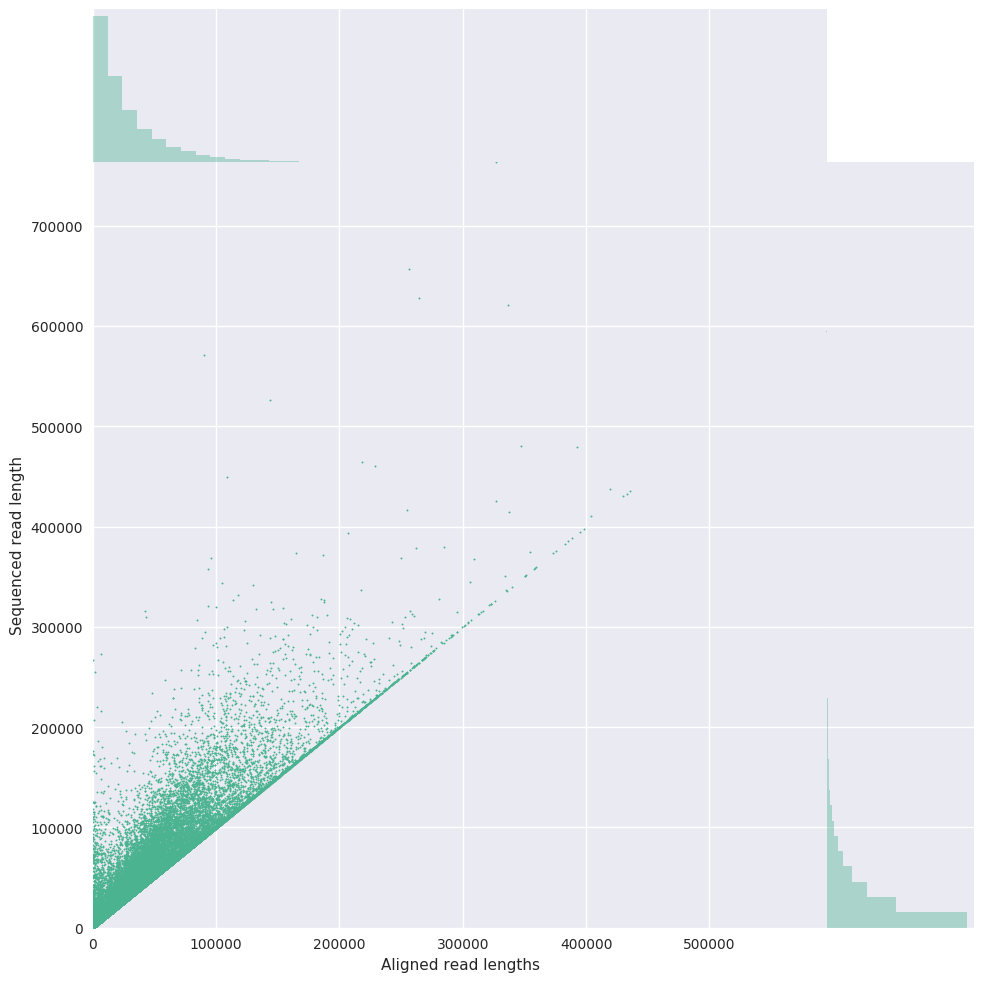


NanoPlot bivariate plot of sequenced reads lengths against aligned read lengths, showing reads not aligning end to end to the reference genome.

## Supplementary Figure S5: NanoComp comparison of throughput in megabases


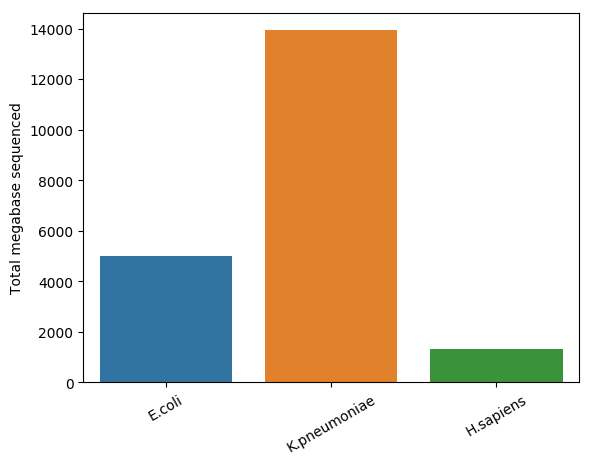


NanoComp bar chart of throughput in megabases per dataset.

## Supplementary Figure S6: NanoComp comparison of quality distribution using violin plots


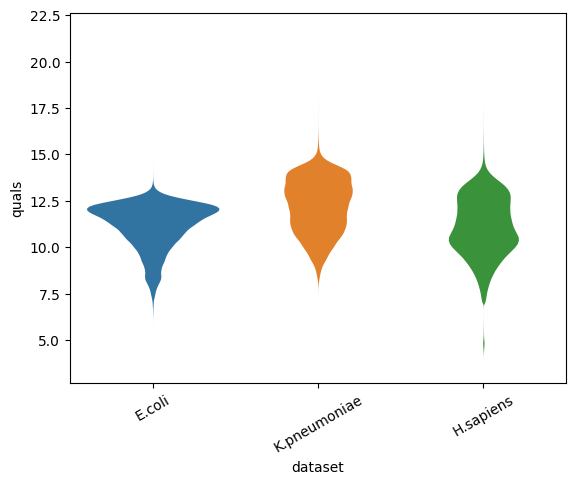


NanoComp violin plot of per read average read quality per dataset.

## Supplementary Figure S7: NanoComp comparison of percent reference identities


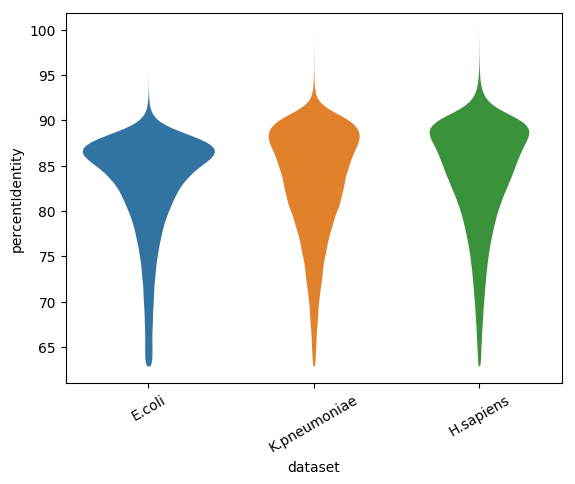


NanoComp violin plot of per read percent reference identity (edit distance scaled to aligned read length) compared to the reference genome.
